# Examining the activity of cefepime-taniborbactam against *Burkholderia cepacia* complex and *Burkholderia gladioli* isolated from cystic fibrosis patients in the United States

**DOI:** 10.1128/aac.00498-23

**Published:** 2023-09-28

**Authors:** Maria F. Mojica, Elise T. Zeiser, Scott A. Becka, John J. LiPuma, David A. Six, Greg Moeck, Krisztina M. Papp-Wallace

**Affiliations:** 1 Department of Molecular Biology and Microbiology, Case Western Reserve University, Cleveland, Ohio, USA; 2 Research Service, Veterans Affairs Northeast Ohio Healthcare System, Cleveland, Ohio, USA; 3 CASE-VA Center for Antimicrobial Resistance and Epidemiology, Cleveland, Ohio, USA; 4 University of Michigan, Ann Arbor, Michigan, USA; 5 Venatorx Pharmaceuticals, Inc., Malvern, Pennsylvania, USA; 6 Department of Medicine, Case Western Reserve University, Cleveland, Ohio, USA; 7 Department of Biochemistry, Case Western Reserve University, Cleveland, Ohio, USA; Shionogi Inc., Florham Park, New Jersey, USA

**Keywords:** β-lactamases, *Burkholderia*, β-lactam, PenA, carbapenemase, β-lactamase inhibitor, cefepime-taniborbactam

## Abstract

The novel clinical-stage β-lactam-β-lactamase inhibitor combination, cefepime-taniborbactam, demonstrates promising activity toward many Gram-negative bacteria producing class A, B, C, and/or D β-lactamases. We tested this combination against a panel of 150 *Burkholderia cepacia* complex (Bcc) and *Burkholderia gladioli* strains. The addition of taniborbactam to cefepime shifted cefepime minimum inhibitory concentrations toward the provisionally susceptible range in 59% of the isolates tested. Therefore, cefepime-taniborbactam possessed similar activity as first-line agents, ceftazidime and trimethoprim-sulfamethoxazole, supporting further development.

## INTRODUCTION

**Fig 1:**
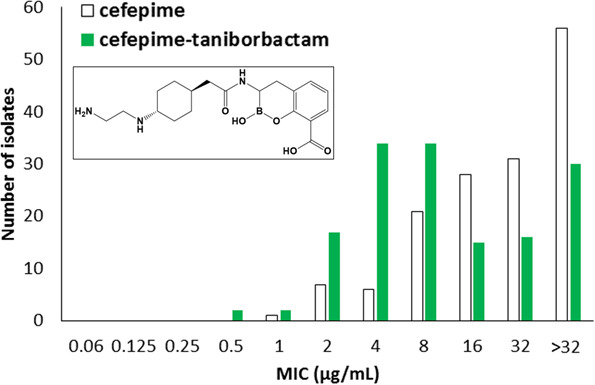
Susceptibility testing results of cefepime alone (white bars) compared to cefepime combined with taniborbactam fixed at 4 µg/mL (green bars) against a panel of 150 clinical isolates of *Burkholderia cepacia* complex and *Burkholderia gladioli*. The chemical structure of taniborbactam (VNRX-5133) is shown in the inset.

Taniborbactam (formerly VNRX-5133) is a novel bicyclic boronic-acid β-lactamase inhibitor (Fig. 1) being developed in combination with cefepime. A phase 3 clinical trial comparing cefepime-taniborbactam to meropenem for the treatment of complicated urinary tract infections and acute pyelonephritis was recently completed (clinicaltrials.gov identifier: NCT03840148). Cefepime-taniborbactam was statistically superior to meropenem for the primary composite endpoint at the test of cure visit (1). Taniborbactam is unique compared to all other clinically available β-lactamase inhibitors as it inhibits class A, B, and C serine-β-lactamases and class D metallo-β-lactamases (2 – 12). Cefepime-taniborbactam demonstrates potent antimicrobial activity against *Pseudomonas aeruginosa*, including strains producing VIM-2 (3, 7, 9 – 11, 13). Cefepime-taniborbactam reduced the bacterial load in lung, urinary tract, and thigh murine infection models caused by cephalosporin-resistant *Klebsiella pneumoniae* and *Escherichia coli* as well as carbapenem-resistant Enterobacterales, including serine and metallo-β-lactamase (MBL) producers (4, 14 – 17).

The *Burkholderia cepacia* complex (Bcc) is a group of >20 related non-fermenting pathogens (18). Bcc and *Burkholderia gladioli* can cause chronic infections in the immunocompromised and those with cystic fibrosis (CF) with 8%–10% of CF patients acquiring Bcc or *B. gladioli* by end stage, leading to increased morbidity and mortality (19, 20). These pathogens are inherently multidrug resistant (MDR); consequently, treatment options are limited (21). β-Lactam antibiotics (e.g., meropenem and ceftazidime) are recommended therapies to treat infections caused by Bcc and *B. gladioli*; however, their utility is declining (22, 23).

Bcc and *B. gladioli* produce chromosomally encoded PenA (class A) and AmpC (class C) β-lactamases (24, 25) that can each vary in their β-lactam hydrolysis spectrum (24 – 27). The PenA1 β-lactamase of *Burkholderia multivorans* ATCC 17616 confers resistance to many β-lactams (e.g., ampicillin, ceftazidime, aztreonam, and imipenem) as well as β-lactam-β-lactamase inhibitor combinations (e.g., ampicillin-clavulanic acid) (26), while the narrow-spectrum AmpC1 contribution to β-lactam resistance is minimal (24). Several β-lactamase inhibitors, including diazabicyclooctane-based (i.e., avibactam, relebactam, and durlobactam) and boronate-based (i.e., vaborbactam) compounds, were found to inactivate the PenA1 carbapenemase of *B. multivorans in vitro* (22, 28 – 30). The combinations of ceftazidime-avibactam, imipenem-relebactam, and sulbactam-durlobactam demonstrated potency against Bcc and *B. gladioli* CF isolates (22, 28, 29).

We evaluated the activity of cefepime-taniborbactam against a panel of 140 Bcc clinical isolates (i.e., species *ambifaria*, *arboris*, *cenocepacia*, *cepacia*, *contaminans*, *diffusa*, *dolosa*, *multivorans*, *pseudomultivorans*, *pyrrocinia*, *seminalis*, *stabilis*, *ubonensis*, and *vietnamiensis*) and 10 *B. gladioli* from the *Burkholderia cepacia* Research Laboratory and Repository (University of Michigan) (22, 25, 31) (Supplementary Material, Tables S1 and S2). *K. pneumoniae* ATCC 700603 carrying *bla*_SHV-18_ and *K. pneumoniae* ATCC BAA-1705 harboring *bla*_KPC_ strains were used as controls for β-lactam and β-lactamase inhibitor integrity; all controls tested within the anticipated quality control ranges (Table S3). Minimum inhibitory concentration (MIC) results were interpreted using Clinical Laboratory Standards Institute (CLSI) breakpoints, where available (32). Cefepime-taniborbactam MIC results were provisionally interpreted using cefepime breakpoints for *P. aeruginosa* of susceptible (≤8 µg/mL), intermediate (16 µg/mL), and resistant (>32 µg/mL) (32). An alternative cefepime-taniborbactam provisional susceptible breakpoint of ≤16 µg/mL is supported by *in vivo* efficacy data from neutropenic murine infection models (thigh, complicated urinary tract, and lung) (15 – 17) and data from safety and pharmacokinetics studies in human volunteers (33, 34). The cefepime-taniborbactam antimicrobial activity was compared to cefepime, meropenem, meropenem-vaborbactam, and levofloxacin, to data published for first-line agents (e.g., ceftazidime and trimethoprim-sulfamethoxazole) and to those recommended by the CLSI for clinical testing (e.g., minocycline) against the Bcc and *B. gladioli* panel (22, 32). Of note, the MICs of all these agents were determined by the agar dilution method, which alongside broth microdilution, are recommended by the CLSI to assess the antimicrobial susceptibility of Bcc isolates (32).

Compared to cefepime alone, the addition of taniborbactam (at 4 µg/mL) shifted the MICs toward the provisionally susceptible range; the MIC_50_ value decreased by fourfold from 32 to 8 µg/mL (Table S1; Fig. 1). The percentage of isolates inhibited at ≤8 µg/mL increased from 23% (cefepime alone) to 59% (cefepime-taniborbactam) and at ≤16 µg/mL increased from 42% to 69%, respectively. A bimodal MIC distribution was apparent for *B. multivorans* and *B. cenocepacia* within this challenge panel (Fig. 1). Moreover, four strains, *B. dolosa* AU29985, *B. multivorans* AU28442, *B. multivorans* AU11772, and *B. multivorans* AU15814, demonstrated properties of extremely drug-resistant strains with high MICs against all antibiotics tested. These observations may be due to altered profiles and/or expression levels of the PenA-like β-lactamases, as we have previously shown significant heterogeneity of PenA within *B. multivorans* as well as changes to the level of induction of *bla*_PenA_ (25, 31). Poirel et al. observed heterogeneity in PenB β-lactamases from *B. cenocepacia* (27). Alterations in the target PBP may play a role as previously observed for *Burkholderia pseudomallei* (35); efflux is also a major resistance determinant in *Burkholderia* species (36).

**Fig 2:**
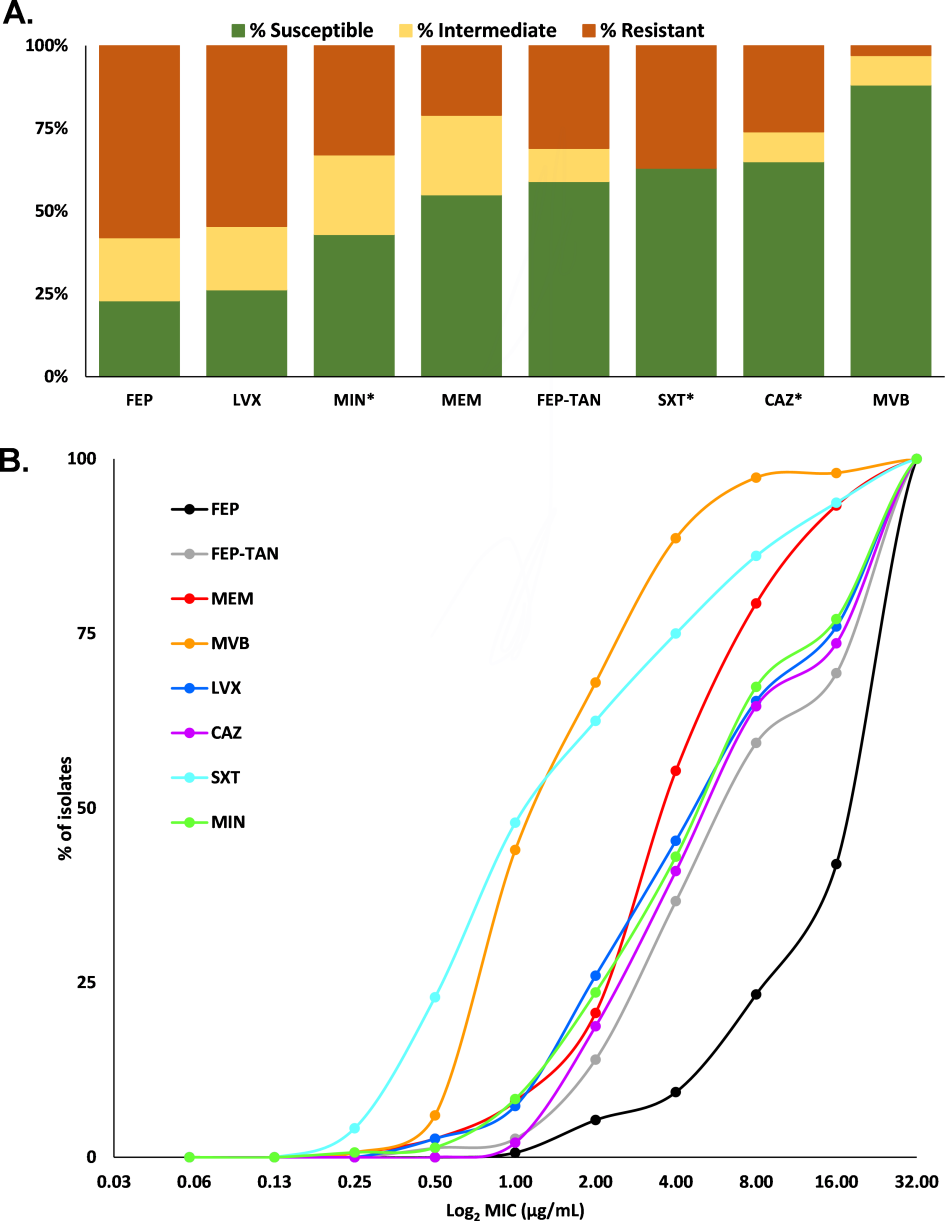
Susceptibility testing comparison for all tested agents and antibiotics recommended by CLSI for clinical testing against Bcc, identified by an asterisk [data previously published (22)]. (A) A bar graph revealing the % susceptible, % intermediate, and % resistant to each antibiotic. MIC interpretations for FEP-TAN are based on FEP breakpoints for *P. aeruginosa*. Taniborbactam was fixed at 4 µg/mL, and vaborbactam was fixed at 8 µg/mL. (B) A line graph representing the percentage of isolates with a specific MIC. FEP, cefepime; LVX, levofloxacin; MIN, minocycline; MEM, meropenem; FEP-TAN, cefepime-taniborbactam; SXT, trimethoprim-sulfamethoxazole; CAZ, ceftazidime; MVB, meropenem-vaborbactam.

Among the comparators, meropenem was the most effective β-lactam against this panel of MDR *Burkholderia* species with MIC_50_ of 4 µg/mL compared to 32 µg/mL for cefepime. The addition of 8 µg/mL of vaborbactam further increased the potency of meropenem with meropenem-vaborbactam yielding an MIC_50_ of 2 µg/mL. Levofloxacin performed similarly to cefepime alone with 26% vs 23% of isolates testing susceptible, respectively. Notably, only 4/150 strains (3%) possessed high MIC values to cefepime, cefepime-taniborbactam, meropenem, meropenem-vaborbactam, and levofloxacin. The rank order of potency by percent susceptibility or percent provisional susceptibility (≤16 µg/mL for cefepime-taniborbactam) against these *Burkholderia* spp. was as follows: meropenem-vaborbactam (89%S) > cefepime-taniborbactam (69%S) > meropenem (55%S) > levofloxacin (26%S) > cefepime (23%S) (Table S1; Fig. 2). Susceptibility profiles did not congregate based on species (Table S2).

The cefepime-taniborbactam antimicrobial susceptibility data were compared to published data for CLSI recommended agents for Bcc with the same strain panel (22, 29, 32). Cefepime-taniborbactam (69% of isolates inhibited at ≤16 µg/mL) was comparable to first-line agents, ceftazidime (64%S) and trimethoprim-sulfamethoxazole (63%S) (Fig. 2). Cefepime-taniborbactam was more active than meropenem, levofloxacin, and minocycline. Cefepime activity against *Burkholderia* species remains to be established. Ceftazidime is the cephalosporin of choice for treating infections caused by *Burkholderia* species. Of this challenge panel, only 36% of isolates were reported to be resistant to ceftazidime (28) compared to cefepime, where 58% were resistant; thus, likely, the cephalosporin partner influences the high MIC_90_ values for cefepime and cefepime-taniborbactam of >32 µg/mL.

The parameters for inhibition of the *B. multivorans* ATCC 17616 carbapenemase PenA1 by taniborbactam were determined and compared to other β-lactamase inhibitors, as described in the Supplementary Material and Methods. Taniborbactam demonstrated potent inactivation of the *B. multivorans* PenA1 with a *K*_i app_ value of 0.4 µM that is comparable to that of avibactam (*K*_i app_ = 0.5 µM) but much lower than relebactam and vaborbactam 3.2 and 38 µM, respectively (Table 1) (22, 29, 30). Moreover, the acylation rate for the bicyclic boronate, taniborbactam was >250-fold higher than for the monocyclic boronate, vaborbactam. The lowest *k*_off_ rate was for taniborbactam. These results demonstrate that the efficacy of cefepime-taniborbactam is largely the result of the taniborbactam inhibition of PenA1 β-lactamase. Taniborbactam and avibactam inhibited PenA1 *in vitro* with relatively similar potencies, while relebactam and vaborbactam exhibited weaker inhibitory kinetic profiles in comparison. In summary, cefepime-taniborbactam will be a welcome addition to the antibiotic arsenal due to its broad-spectrum activity against Enterobacterales and *P. aeruginosa* producing class A, B, C, and D β-lactamases. With broader analyses, cefepime-taniborbactam may also be valuable to treat infections caused by *Burkholderia* species.

**TABLE 1:** Steady-state inhibitor kinetic values against *B. multivorans* PenA1^*a*^

Parameter	Taniborbactam	Avibactam (22)	Relebactam (29)	Vaborbactam (30)
*K*_i app_ (nM)	400 ± 40	500 ± 50	3,200 ± 300	38,000 ± 4000
*k*_2_/*K* (M^−1^s^−1^)	8.8 ± 1.1 × 10^4^	2 ± 1 × 10^6^	7.2 ± 2.0 × 10^3^	3.4 ± 0.1 × 10^2^
*k*_off_ (s^−1^)	2 ± 1 × 10^−4^	2 ± 1 × 10^−3^	1.4 ± 0.5 × 10^−2^	n/d^*b*^
*t*_1/2_ (min)	58	5.8	0.83	n/d

^*a*^ Individual data points were collected in triplicate, while all experiments were completed at a minimum in duplicate.

^*b*^ n/d, not determined.

## AFTER EPUB

*[This article was published on 28 September 2023 with a standard copyright line ("Copyright © 2023 American Society for Microbiology. All Rights Reserved."). The authors elected to pay for open access for the article after publication, necessitating replacement of the original copyright line, and this change was made on 6 October 2023.]*

## SUPPLEMENTAL MATERIAL

Supplemental Material aac.00498-23-s0001.docx Supplementary Material and Methods, and Supplementary Tables.

ASM does not own the copyrights to Supplemental Material that may be linked to, or accessed through, an article. The authors have granted ASM a non-exclusive, world-wide license to publish the Supplemental Material files. Please contact the corresponding author directly for reuse.
