# Supplementary material for: Examining the activity of cefepime-taniborbactam against Burkholderia cepacia complex and Burkholderia gladioli isolated from cystic fibrosis patients in the United States
Source: Antimicrob Agents Chemother. 2023 Sep 28;67(11):e00498-23. doi: 10.1128/aac.00498-23 (PMC10648927; doi:10.1128/aac.00498-23)
Supplement: Supplemental Material — Supplementary Material and Methods, and Supplementary Tables. [file aac.00498-23-s0001.docx]

Supplementary Materials and Methods

***Antibiotics.*** Cefepime, meropenem, and levofloxacin were purchased from Sigma; taniborbactam (VNRX-5133) and vaborbactam were provided by Venatorx.

***Bacterial Strains.*** 150 MDR clinical strains, including 140 Bcc (14 different species) and 10 *B. gladioli* were obtained from the *Burkholderia cepacia* Research Laboratory and Repository (University of Michigan) (21, 24, 31). These 150 isolates were recovered from respiratory specimens from 150 individuals with CF receiving care in 68 cities in 36 states in the US. Each Bcc isolate was identified to the species level by using species-specific polymerase chain reaction, *recA* RFLP and/or DNA sequencing of the *recA* gene (32, 33). If the species could not be determined, the isolate is listed as Bcc Indeterminate.

***In vitro Susceptibility Test Methods.*** Minimum inhibitory concentrations (MICs) for the bacterial isolates were determined by the cation-adjusted Mueller-Hinton (MH) agar dilution method. The MIC measurements were performed using a Steers^TM^ Replicator that delivered 10 µL at 10^4^ colony forming units (CFUs) of overnight culture grown in MH broth at 37 °C with shaking and diluted into fresh MH broth. Taniborbactam was tested at 4 µg/mL in combination with increasing concentrations of cefepime. Vaborbactam was tested at 8 µg/mL with increasing concentrations of meropenem. MIC results were interpreted using CLSI breakpoints, where available (30). For comparative purposes only, cefepime-taniborbactam MIC results were provisionally interpreted using cefepime breakpoints for *P. aeruginosa* of susceptible (≤8 µg/mL), intermediate (16 µg/mL), and resistant (>32 µg/mL) (30). An alternative cefepime-taniborbactam provisional susceptible breakpoint of ≤16 µg/mL is supported by *in vivo* efficacy data from neutropenic murine infection models (thigh, complicated urinary tract, lung) (14-16) and data from safety and pharmacokinetics studies in human volunteers (34, 35). *K. pneumoniae* ATCC 700603 carrying *bla*_SHV-18_ and *K. pneumoniae* ATCC BAA-1705 with *bla*_KPC_ strains were used as controls for β-lactam and β-lactamase inhibitor integrity.

***Enzyme Expression, Purification, and Steady-state Kinetics.*** *E*. *coli* DE3 Origami 2 cells carrying pGEX-6p2 *bla*_penA1_ were used for protein expression and purification, as previously described (25, 36). Briefly, cells were grown in Super Optimal Broth, and then isopropyl-β-d-1-thiogalactopyranoside was added to induce expression. Cells were pelleted and frozen at -80 °C. Subsequently, the cells were lysed, and the β-lactamase was purified and verified by electrospray-ionization mass spectrometry, as previously described (25, 36). Kinetic parameters were obtained as described below; individual data points were obtained in triplicate, while each experiment was conducted in at least duplicate.

The apparent *K*_i_ (*K*_i app_) value, *k*_2_/*K* value or acylation rate, and *k*_off_ for taniborbactam and PenA1 were obtained using an Agilent 8453 Diode Array spectrophotometer in 10 mM phosphate-buffered saline (PBS), pH 7.4 at room temperature using previously described methods (37, 38).

*K*_i app_ was determined for PenA1 using a direct competition assay under steady-state conditions. PenA1 was maintained at 5 nM, while the taniborbactam concentration was varied. Nitrocefin was used as the reporter substrate at a fixed concentration of 100 µM. The PenA1 β-lactamase, taniborbactam, and nitrocefin were mixed manually, and the initial reaction velocity was monitored. Data were linearized by plotting the inverse initial reaction velocities (1/*v*_0_) versus inhibitor concentration (I). *K*_i_ _app_ was determined by dividing the value for the y-intercept by the slope of line and accounting for the use of nitrocefin as a reporter.

To obtain the acylation rate *k*_2_/*K*, progress curves were obtained by mixing PenA1 at 5 nM with increasing concentrations of taniborbactam using nitrocefin at 100 µM as a reporter substrate. Progress curves were fit to Equation 1 to obtain *k*_obs_ values.

Here, *V*_f_ is final velocity, *V*_0_ is initial velocity, and A_0_ is initial absorbance at λ = 482 nm. The data were plotted as *k_obs_* vs [taniborbactam]. The *k*_2_/*K* value was obtained by correcting the value obtained for the slope of the line for the use of nitrocefin as an indicator substrate.

The off-rate, *k*_off_, of taniborbactam for PenA1 was determined by incubating 1 µM of PenA1 with 4 µM taniborbactam (10× *K*_i app_) for 5 min at room temperature, diluting the mixture to a final PenA1 concentration of 1 nM, and adding 100 µM nitrocefin. Progress curves measuring nitrocefin hydrolysis were collected for 1 h and the data were fit to exponential Equation 2 to obtain *k*_off_. PenA1 alone and taniborbactam alone were used as controls.

$\mathbf{y}\boldsymbol{=}\mathbf{A}_{\boldsymbol{0}}\mathbf{e}^{\boldsymbol{k}_{\mathbf{off}}\mathbf{x}}$ **Eq. 2**

**Supplementary results**

Table S1 Agar dilution susceptibility testing results for 150 Bcc and *B*. *gladioli* strains. MIC data is present in µg/mL.

| **Strains** | **cefepime** | **cefepime-taniborbactam*** | **meropenem** | **meropenem-vaborbactam*** | **levofloxacin** |
| --- | --- | --- | --- | --- | --- |
| MIC_50_ | 32 | 8 | 4 | 2 | 8 |
| MIC_90_ | >32 | >32 | 16 | 8 | >32 |
|  | | | | | |
| No. Susceptible (%) | 35 (23%) | 89 (59%) | 83 (55%) | 133 (89%) | 39 (26%) |
| No. Intermediate (%) | 28 (19%) | 15 (10%) | 36 (24%) | 14 (9%) | 30 (20%) |
| No. Resistant (%) | 87 (58%) | 46 (31%) | 31 (21%) | 3 (2%) | 81 (54%) |
|  | | | | | |
| *B. ambifaria* AU5203 | 16 | 4 | 4 | 1 | >32 |
| *B. ambifaria* AU19862 | 16 | 16 | 2 | 1 | 8 |
| *B. ambifaria* AU20319 | >32 | 32 | 4 | 0.5 | 1 |
| *B. ambifaria* AU11161 | >32 | 32 | 8 | 2 | 32 |
| *B. arboris* AU14372 | >32 | 16 | 16 | 4 | 8 |
| *B. arboris* AU24192 | 32 | 8 | 8 | 1 | 4 |
| *B. cenocepacia* AU3663 | 32 | 4 | 4 | 2 | 16 |
| *B. cenocepacia* AU10321 | >32 | 32 | 16 | 8 | >32 |
| *B. cenocepacia* AU141094 | >32 | >32 | 16 | 8 | >32 |
| *B. cenocepacia* AU0133 | 16 | 8 | 4 | 2 | 4 |
| *B. cenocepacia* AU0756 | >32 | >32 | 32 | 8 | >32 |
| *B. cenocepacia* AU8635 | >32 | 8 | 16 | 4 | >32 |
| *B. cenocepacia* AU9215 | >32 | >32 | 16 | 8 | 32 |
| *B. cenocepacia* AU9512 | 32 | 16 | 4 | 4 | 16 |
| *B. cenocepacia* AU9710 | 16 | 2 | 4 | 1 | 4 |
| *B. cenocepacia* AU11339 | 16 | 4 | 2 | 1 | 2 |
| *B. cenocepacia* AU12265 | 16 | 8 | 4 | 2 | 4 |
| *B. cenocepacia* AU12571 | 16 | 8 | 2 | 1 | 8 |
| *B. cenocepacia* AU12659 | 16 | 2 | 4 | 2 | 1 |
| *B. cenocepacia* AU13262 | >32 | 4 | 4 | 1 | 4 |
| *B. cenocepacia* AU16448 | 16 | 4 | 4 | 1 | 8 |
| *B. cenocepacia* AU17796 | 16 | 8 | 4 | 2 | 8 |
| *B. cenocepacia* AU19276 | 8 | 2 | 2 | 1 | 0.5 |
| *B. cenocepacia* AU19530 | >32 | 16 | 8 | 2 | 16 |
| *B. cenocepacia* AU20146 | 16 | 4 | 4 | 1 | 2 |
| *B. cenocepacia* AU20545 | 8 | 2 | 2 | 1 | 2 |
| *B. cenocepacia* AU20902 | 16 | 4 | 4 | 2 | 4 |
| *B. cenocepacia* AU21083 | 16 | 4 | 4 | 1 | 2 |
| *B. cenocepacia* AU28111 | 32 | 4 | 4 | 1 | 4 |
| *B. cenocepacia* AU29265 | 16 | 2 | 2 | 0.5 | 2 |
| *B. cenocepacia* AU24362 | >32 | >32 | 16 | 4 | 8 |
| *B. cenocepacia* AU0583 | 32 | 2 | 4 | 1 | >32 |
| *B. cenocepacia* AU6550 | 32 | 16 | 8 | 2 | 8 |
| *B. cenocepacia* AU9292 | 32 | 8 | 4 | 1 | 32 |
| *B. cenocepacia* AU14093 | >32 | >32 | 8 | 2 | >32 |
| *B. cenocepacia* AU22509 | >32 | 16 | 8 | 2 | 32 |
| *B. cenocepacia* AU14381 | 32 | 16 | 4 | 2 | >32 |
| *B. cenocepacia* AU19684 | >32 | 32 | 8 | 4 | >32 |
| *B. cenocepacia* AU20998 | >32 | 8 | 8 | 1 | >32 |
| *B. cepacia* AU0108 | 8 | 2 | 4 | 1 | 2 |
| *B. cepacia* AU0329 | >32 | 8 | 2 | 1 | >32 |
| *B. cepacia* AU1555 | >32 | 32 | 8 | 4 | 16 |
| *B. cepacia* AU11420 | >32 | 16 | 8 | 4 | 4 |
| *B. cepacia* AU13163 | 16 | 2 | 4 | 1 | 2 |
| *B. cepacia* AU13354 | >32 | 32 | 8 | 2 | 8 |
| *B. cepacia* AU15249 | 32 | 4 | 4 | 1 | 4 |
| *B. cepacia* AU15677 | 32 | 4 | 4 | 1 | 4 |
| *B. cepacia* AU16396 | 32 | 8 | 8 | 2 | >32 |
| *B. cepacia* AU17630 | >32 | 8 | 4 | 1 | 4 |
| *B. cepacia* AU19265 | 32 | 8 | 4 | 1 | 4 |
| *B. cepacia* AU22213 | 32 | 4 | 4 | 1 | 2 |
| *B. cepacia* AU23707 | 32 | 16 | 8 | 4 | 32 |
| *B. cepacia* AU27816 | 16 | 2 | 4 | 1 | 4 |
| *B. cepacia* AU29493 | >32 | 8 | 8 | 2 | 8 |
| *B. contaminans* AU15669 | >32 | 8 | 16 | 4 | 8 |
| *B. contaminans* AU21811 | >32 | >32 | 16 | 4 | 8 |
| *B. contaminans* AU2244 | 16 | 4 | 4 | 1 | 4 |
| *B. contaminans* AU22662 | 16 | 4 | 4 | 1 | 4 |
| *B. contaminans* AU25403 | 8 | 0.5 | 2 | 1 | 2 |
| *B. contaminans* AU17641 | 32 | 4 | 4 | 1 | 4 |
| *B. contaminans* AU20979 | >32 | >32 | 8 | 4 | 32 |
| *B. diffusa* AU19637 | 32 | 4 | 2 | 0.5 | 2 |
| *B. dolosa* AU9336 | >32 | 8 | 8 | 2 | 32 |
| *B. dolosa* AU12872 | >32 | >32 | 32 | 8 | >32 |
| *B. dolosa* AU29021 | >32 | 32 | 8 | 2 | 4 |
| *B. dolosa* AU29985 | >32 | >32 | >32 | 32 | >32 |
| *B. gladioli* AU0032 | >32 | >32 | 2 | 2 | 8 |
| *B. gladioli* AU1009 | 16 | 4 | 1 | 1 | 0.5 |
| *B. gladioli* AU21101 | 16 | 4 | 1 | 1 | 1 |
| *B. gladioli* AU27927 | 8 | 4 | 1 | 0.5 | 1 |
| *B. gladioli* AU28659 | 32 | 32 | 2 | 2 | 2 |
| *B. gladioli* AU29223 | 16 | 4 | 1 | 1 | 0.5 |
| *B. gladioli* AU30473 | 32 | 16 | 4 | 2 | 2 |
| *B. gladioli* AU26456 | 4 | 4 | 1 | 1 | 0.5 |
| *B. gladioli* AU16341 | >32 | >32 | 8 | 8 | 4 |
| *B. gladioli* AU29541 | 32 | 32 | 8 | 8 | 8 |
| *B. multivorans* AU28442 | >32 | >32 | >32 | >32 | 16 |
| *B. multivorans* AU4507 | 8 | 4 | 8 | 4 | 8 |
| *B. multivorans* AU10398 | >32 | >32 | 8 | 4 | 16 |
| *B. multivorans* AU10897 | 32 | 16 | 8 | 4 | 16 |
| *B. multivorans* AU14786 | >32 | >32 | 16 | 4 | >32 |
| *B. multivorans* AU19518 | 32 | 8 | 16 | 4 | 32 |
| *B. multivorans* AU21015 | >32 | >32 | 16 | 8 | 32 |
| *B. multivorans* AU21596 | >32 | >32 | 32 | 8 | >32 |
| *B. multivorans* AU22436 | 8 | 8 | 8 | 2 | >32 |
| *B. multivorans* AU23365 | >32 | >32 | 8 | 2 | 8 |
| *B. multivorans* AU23690 | 32 | 16 | 4 | 2 | >32 |
| *B. multivorans* AU23919 | 32 | 2 | 4 | 2 | 2 |
| *B. multivorans* AU23995 | >32 | 4 | 8 | 2 | 4 |
| *B. multivorans* AU24277 | >32 | >32 | 16 | 4 | 32 |
| *B. multivorans* AU25057 | 8 | 4 | 4 | 2 | 8 |
| *B. multivorans* AU26250 | 4 | 4 | 2 | 1 | 2 |
| *B. multivorans* AU18096 | 2 | 4 | 1 | 1 | 4 |
| *B. multivorans* AU28069 | 8 | 8 | 4 | 1 | 4 |
| *B. multivorans* AU30438 | 16 | 8 | 4 | 1 | 4 |
| *B. multivorans* AU11358 | 8 | 8 | 8 | 2 | 2 |
| *B. multivorans* AU17534 | >32 | >32 | 16 | 4 | ND |
| *B. multivorans* AU17135 | 4 | 8 | 4 | 2 | 2 |
| *B. multivorans* AU14364 | >32 | 32 | 4 | 4 | 32 |
| *B. multivorans* AU13919 | 8 | 8 | 8 | 2 | 2 |
| *B. multivorans* AU11772 | >32 | >32 | 16 | 16 | 8 |
| *B. multivorans* AU14371 | 8 | 32 | 4 | 1 | 2 |
| *B. multivorans* AU10086 | 32 | >32 | 32 | 4 | >32 |
| *B. multivorans* AU29198 | >32 | >32 | 16 | 8 | 8 |
| *B. multivorans* AU14328 | 8 | 8 | 4 | 1 | 2 |
| *B. multivorans* AU11204 | 4 | 8 | 2 | 1 | 2 |
| *B. multivorans* AU19729 | >32 | >32 | 16 | 4 | 16 |
| *B. multivorans* AU12481 | 2 | 1 | 2 | 1 | 4 |
| *B. multivorans* AU22892 | 2 | 2 | 4 | 1 | 2 |
| *B. multivorans* AU27706 | 1 | 0.5 | 0.5 | 0.5 | 1 |
| *B. multivorans* AU30050 | 2 | 1 | 2 | 1 | 1 |
| *B. multivorans* AU25543 | 4 | 4 | 1 | 1 | 2 |
| *B. multivorans* AU11233 | >32 | >32 | 8 | 4 | 16 |
| *B. multivorans* AU19564 | 16 | 8 | 4 | 1 | 8 |
| *B. multivorans* AU15814 | 32 | 32 | >32 | >32 | 16 |
| *B. multivorans* AU23668 | 4 | 2 | 2 | 0.5 | 2 |
| *B. multivorans* AU15954 | 8 | 4 | 4 | 1 | 2 |
| *B. multivorans* AU20929 | 8 | 4 | 8 | 1 | 4 |
| *B. multivorans* AU30760 | 8 | 4 | 2 | 1 | 2 |
| *B. multivorans* AU25626 | 8 | 8 | >32 | 32 | 4 |
| *B. multivorans* AU30441 | 2 | 2 | 8 | 1 | 2 |
| *B. multivorans* AU17545 | 16 | 8 | 2 | 1 | 8 |
| *B. multivorans* AU19659 | >32 | >32 | 32 | 8 | 16 |
| *B. multivorans* AU10047 | 32 | 8 | 16 | 4 | 8 |
| *B. multivorans* AU16734 | 16 | 8 | 16 | 4 | 8 |
| *B. pseudomultivorans* AU19682 | >32 | 16 | 8 | 4 | 16 |
| *B. pyrrocinia* AU1114 | 8 | 4 | 4 | 1 | >32 |
| *B. pyrrocinia* AU4348 | 32 | 4 | 4 | 1 | >32 |
| *B. seminalis* AU14842 | 16 | 16 | 4 | 2 | 4 |
| *B. stabilis* AU9035 | 32 | 16 | 8 | 4 | 16 |
| *B. stabilis* AU10235 | >32 | >32 | 16 | 8 | 16 |
| *B. ubonensis* AU7314 | >32 | >32 | 32 | 8 | 4 |
| *B. vietnamiensis* AU19457 | >32 | 8 | 4 | 2 | 16 |
| *B. vietnamiensis* AU21549 | 2 | 2 | 0.5 | 0.5 | 4 |
| *B. vietnamiensis* AU3997 | 8 | 8 | 1 | 1 | 8 |
| *B. vietnamiensis* AU5003 | >32 | 8 | 4 | 1 | 8 |
| *B. vietnamiensis* AU10214 | 8 | 4 | 0.5 | 0.5 | 8 |
| *B. vietnamiensis* AU3578 | >32 | >32 | 4 | 4 | 8 |
| *B. vietnamiensis* AU26096 | 32 | 32 | 8 | 4 | >32 |
| *B. vietnamiensis* AU28056 | 16 | 32 | 4 | 2 | 8 |
| *B. vietnamiensis* AU28891 | 8 | 8 | 2 | 2 | 8 |
| *B. vietnamiensis* AU30387 | 2 | 2 | 0.25 | 0.25 | 2 |
| Bcc Indeterminate AU9162 | 32 | 4 | 4 | 1 | 1 |
| Bcc Indeterminate AU12560 | 32 | 8 | 4 | 2 | 8 |
| Bcc Indeterminate AU12848 | >32 | >32 | 8 | 2 | 8 |
| Bcc Indeterminate AU14915 | >32 | 16 | 16 | 4 | 16 |
| Bcc Indeterminate AU14962 | >32 | 2 | 8 | 1 | 2 |
| Bcc Indeterminate AU18117 | >32 | 32 | 8 | 4 | >32 |
| Bcc Indeterminate AU19076 | 16 | 2 | 4 | 1 | 4 |
| Bcc Indeterminate AU19821 | >32 | >32 | 16 | 2 | 32 |

| Breakpoints for cefepime (susceptible (S) ≤ 8 µg/mL; intermediate = 16 µg/mL; resistant (R) ≥ 32 µg/mL) for *P*. *aeruginosa* were used to assign phenotypes for cefepime and the combination with taniborbactam. Breakpoints for meropenem (susceptible (S) ≤ 4 µg/mL; intermediate (I) = 8 µg/mL; resistant (R) ≥ 16 µg/mL) were used to assign phenotypes to meropenem-vaborbactam. Breakpoints for levofloxacin were as follows (susceptible (S) ≤ 2 µg/mL; intermediate = 4 µg/mL; resistant (R) ≥ 8 µg/mL). NA: not available. ND: not determinable. *Taniborbactam was tested at a fixed concentration of 4 µg/mL, while vaborbactam was tested at a fixed concentration of 8 µg/mL. |
| --- |

Table S2 Agar dilution susceptibility testing results grouped by species.

| **Species** | | **Cefepime MIC (µg/mL)** | | | | | | | | | | | | | | | | | |
| --- | --- | --- | --- | --- | --- | --- | --- | --- | --- | --- | --- | --- | --- | --- | --- | --- | --- | --- | --- |
|  | | | | **0.06** | **0.125** | **0.25** | **0.5** | **1** | **2** | **4** | **8** | **16** | **32** | **>32** | **R** | **I** | **S** | |  |
| *B. ambifaria* (4) | | | | 0 | 0 | 0 | 0 | 0 | 0 | 0 | 0 | 2 | 0 | 2 | 2 | 2 | 0 | |  |
| *B. arboris* (2) | | | | 0 | 0 | 0 | 0 | 0 | 0 | 0 | 0 | 0 | 1 | 1 | 2 | 0 | 0 | |  |
| *B. cenocepacia* (33) | | | | 0 | 0 | 0 | 0 | 0 | 0 | 0 | 2 | 12 | 7 | 12 | 19 | 12 | 2 | |  |
| *B. cepacia* (15) | | | | 0 | 0 | 0 | 0 | 0 | 0 | 0 | 1 | 2 | 6 | 6 | 12 | 2 | 1 | |  |
| *B. contaminans* (7) | | | | 0 | 0 | 0 | 0 | 0 | 0 | 0 | 1 | 2 | 1 | 3 | 4 | 2 | 1 | |  |
| *B. diffusa* (1) | | | | 0 | 0 | 0 | 0 | 0 | 0 | 0 | 0 | 0 | 1 | 0 | 1 | 0 | 0 | |  |
| *B. dolosa* (4) | | | | 0 | 0 | 0 | 0 | 0 | 0 | 0 | 0 | 0 | 0 | 4 | 4 | 0 | 0 | |  |
| *B. gladioli* (10) | | | | 0 | 0 | 0 | 0 | 0 | 0 | 1 | 1 | 3 | 3 | 2 | 5 | 3 | 2 | |  |
| *B. multivorans* (49) | | | | 0 | 0 | 0 | 0 | 1 | 5 | 5 | 12 | 4 | 7 | 15 | 22 | 4 | 23 | |  |
| *B. pseudomultivorans* (1) | | | | 0 | 0 | 0 | 0 | 0 | 0 | 0 | 0 | 0 | 0 | 1 | 1 | 0 | 0 | |  |
| *B. pyrrocinia* (2) | | | | 0 | 0 | 0 | 0 | 0 | 0 | 0 | 1 | 0 | 1 | 0 | 1 | 0 | 1 | |  |
| *B. seminalis* (1) | | | | 0 | 0 | 0 | 0 | 0 | 0 | 0 | 0 | 1 | 0 | 0 | 0 | 1 | 0 | |  |
| *B. stabilis* (2) | | | | 0 | 0 | 0 | 0 | 0 | 0 | 0 | 0 | 0 | 1 | 1 | 2 | 0 | 0 | |  |
| *B. ubonensis* (1) | | | | 0 | 0 | 0 | 0 | 0 | 0 | 0 | 0 | 0 | 0 | 1 | 1 | 0 | 0 | |  |
| *B. vietnamiensis* (10) | | | | 0 | 0 | 0 | 0 | 0 | 2 | 0 | 3 | 1 | 1 | 3 | 4 | 1 | 5 | |  |
| Bcc Indeterminate (8) | | | | 0 | 0 | 0 | 0 | 0 | 0 | 0 | 0 | 1 | 2 | 5 | 7 | 1 | 0 | |  |
| **Total (150)** | | | | **0** | **0** | **0** | **0** | **1** | **7** | **6** | **21** | **28** | **31** | **56** | **87** | **28** | **35** | |  |
| **Species** | **Cefepime-taniborbactam* MIC (µg/mL)** | | | | | | | | | | | | | | | | |  |  |
|  | | | | **0.06** | **0.125** | **0.25** | **0.5** | **1** | **2** | **4** | **8** | **16** | **32** | **>32** | **R** | **I** | **S** | |  |
| *B. ambifaria* (4) | | | | 0 | 0 | 0 | 0 | 0 | 0 | 1 | 0 | 1 | 2 | 0 | 2 | 1 | 1 | |  |
| *B. arboris* (2) | | | | 0 | 0 | 0 | 0 | 0 | 0 | 0 | 1 | 1 | 0 | 0 | 0 | 1 | 1 | |  |
| *B. cenocepacia* (33) | | | | 0 | 0 | 0 | 0 | 0 | 6 | 8 | 7 | 5 | 2 | 5 | 7 | 5 | 21 | |  |
| *B. cepacia* (15) | | | | 0 | 0 | 0 | 0 | 0 | 3 | 4 | 4 | 2 | 2 | 0 | 2 | 2 | 11 | |  |
| *B. contaminans* (7) | | | | 0 | 0 | 0 | 1 | 0 | 0 | 3 | 1 | 0 | 0 | 2 | 2 | 0 | 5 | |  |
| *B. diffusa* (1) | | | | 0 | 0 | 0 | 0 | 0 | 0 | 1 | 0 | 0 | 0 | 0 | 0 | 0 | 1 | |  |
| *B. dolosa* (4) | | | | 0 | 0 | 0 | 0 | 0 | 0 | 0 | 1 | 0 | 1 | 2 | 3 | 0 | 1 | |  |
| *B. gladioli* (10) | | | | 0 | 0 | 0 | 0 | 0 | 0 | 5 | 0 | 1 | 2 | 2 | 4 | 1 | 5 | |  |
| *B. multivorans* (49) | | | | 0 | 0 | 0 | 1 | 2 | 4 | 9 | 14 | 2 | 3 | 14 | 17 | 2 | 30 | |  |
| *B. pseudomultivorans* (1) | | | | 0 | 0 | 0 | 0 | 0 | 0 | 0 | 0 | 1 | 0 | 0 | 0 | 1 | 0 | |  |
| *B. pyrrocinia* (2) | | | | 0 | 0 | 0 | 0 | 0 | 0 | 2 | 0 | 0 | 0 | 0 | 0 | 0 | 2 | |  |
| *B. seminalis* (1) | | | | 0 | 0 | 0 | 0 | 0 | 0 | 0 | 0 | 1 | 0 | 0 | 0 | 1 | 0 | |  |
| *B. stabilis* (2) | | | | 0 | 0 | 0 | 0 | 0 | 0 | 0 | 0 | 1 | 0 | 1 | 1 | 1 | 0 | |  |
| *B. ubonensis* (1) | | | | 0 | 0 | 0 | 0 | 0 | 0 | 0 | 0 | 0 | 0 | 1 | 1 | 0 | 0 | |  |
| *B. vietnamiensis* (10) | | | | 0 | 0 | 0 | 0 | 0 | 2 | 1 | 4 | 0 | 2 | 1 | 3 | 0 | 7 | |  |
| Bcc Indeterminate (8) | | | | 0 | 0 | 0 | 0 | 0 | 2 | 1 | 1 | 1 | 1 | 2 | 3 | 1 | 4 | |  |
| **Total (150)** | | | | **0** | **0** | **0** | **2** | **2** | **17** | **35** | **33** | **16** | **15** | **30** | **45** | **16** | **89** | |  |
| **Species** | | | **Meropenem MIC (µg/mL)** | | | | | | | | | | | | | | | | |
|  | | | | **0.06** | **0.125** | **0.25** | **0.5** | **1** | **2** | **4** | **8** | **16** | **32** | **>32** | **R** | **I** | **S** | |  |
| *B. ambifaria* (4) | | | | 0 | 0 | 0 | 0 | 0 | 1 | 2 | 1 | 0 | 0 | 0 | 0 | 1 | 3 | |  |
| *B. arboris* (2) | | | | 0 | 0 | 0 | 0 | 0 | 0 | 0 | 1 | 1 | 0 | 0 | 1 | 1 | 0 | |  |
| *B. cenocepacia* (33) | | | | 0 | 0 | 0 | 0 | 0 | 5 | 16 | 6 | 5 | 1 | 0 | 6 | 6 | 21 | |  |
| *B. cepacia* (15) | | | | 0 | 0 | 0 | 0 | 0 | 1 | 8 | 6 | 0 | 0 | 0 | 0 | 6 | 9 | |  |
| *B. contaminans* (7) | | | | 0 | 0 | 0 | 0 | 0 | 1 | 3 | 1 | 2 | 0 | 0 | 2 | 1 | 4 | |  |
| *B. diffusa* (1) | | | | 0 | 0 | 0 | 0 | 0 | 1 | 0 | 0 | 0 | 0 | 0 | 0 | 0 | 1 | |  |
| *B. dolosa* (4) | | | | 0 | 0 | 0 | 0 | 0 | 0 | 0 | 2 | 0 | 1 | 1 | 2 | 2 | 0 | |  |
| *B. gladioli* (10) | | | | 0 | 0 | 0 | 0 | 5 | 2 | 1 | 2 | 0 | 0 | 0 | 0 | 2 | 8 | |  |
| *B. multivorans* (49) | | | | 0 | 0 | 0 | 1 | 2 | 7 | 12 | 11 | 10 | 3 | 3 | 16 | 11 | 22 | |  |
| *B. pseudomultivorans* (1) | | | | 0 | 0 | 0 | 0 | 0 | 0 | 0 | 1 | 0 | 0 | 0 | 0 | 1 | 0 | |  |
| *B. pyrrocinia* (2) | | | | 0 | 0 | 0 | 0 | 0 | 0 | 2 | 0 | 0 | 0 | 0 | 0 | 0 | 2 | |  |
| *B. seminalis* (1) | | | | 0 | 0 | 0 | 0 | 0 | 0 | 1 | 0 | 0 | 0 | 0 | 0 | 0 | 1 | |  |
| *B. stabilis* (2) | | | | 0 | 0 | 0 | 0 | 0 | 0 | 0 | 1 | 1 | 0 | 0 | 1 | 1 | 0 | |  |
| *B. ubonensis* (1) | | | | 0 | 0 | 0 | 0 | 0 | 0 | 0 | 0 | 0 | 1 | 0 | 1 | 0 | 0 | |  |
| *B. vietnamiensis* (10) | | | | 0 | 0 | 1 | 2 | 1 | 1 | 4 | 1 | 0 | 0 | 0 | 0 | 1 | 9 | |  |
| Bcc Indeterminate (8) | | | | 0 | 0 | 0 | 0 | 0 | 0 | 3 | 3 | 2 | 0 | 0 | 2 | 3 | 3 | |  |
| **Total (150)** | | | | **0** | **0** | **1** | **3** | **8** | **19** | **52** | **36** | **21** | **6** | **4** | **31** | **36** | **83** | |  |
| **Species** | | | **Meropenem-Vaborbactam MIC (µg/mL)** | | | | | | | | | | | | | | | | |
|  | | | | **0.06** | **0.125** | **0.25** | **0.5** | **1** | **2** | **4** | **8** | **16** | **32** | **>32** | **R** | **I** | **S** | |  |
| *B. ambifaria* (4) | | | | 0 | 0 | 0 | 1 | 2 | 1 | 0 | 0 | 0 | 0 | 0 | 0 | 0 | 4 | |  |
| *B. arboris* (2) | | | | 0 | 0 | 0 | 0 | 1 | 0 | 1 | 0 | 0 | 0 | 0 | 0 | 0 | 2 | |  |
| *B. cenocepacia* (33) | | | | 0 | 0 | 0 | 1 | 13 | 11 | 4 | 4 | 0 | 0 | 0 | 0 | 4 | 29 | |  |
| *B. cepacia* (15) | | | | 0 | 0 | 0 | 0 | 9 | 3 | 3 | 0 | 0 | 0 | 0 | 0 | 0 | 15 | |  |
| *B. contaminans* (7) | | | | 0 | 0 | 0 | 0 | 4 | 0 | 3 | 0 | 0 | 0 | 0 | 0 | 0 | 7 | |  |
| *B. diffusa* (1) | | | | 0 | 0 | 0 | 1 | 0 | 0 | 0 | 0 | 0 | 0 | 0 | 0 | 0 | 1 | |  |
| *B. dolosa* (4) | | | | 0 | 0 | 0 | 0 | 0 | 2 | 0 | 1 | 0 | 1 | 0 | 1 | 1 | 2 | |  |
| *B. gladioli* (10) | | | | 0 | 0 | 0 | 1 | 4 | 3 | 0 | 2 | 0 | 0 | 0 | 0 | 2 | 8 | |  |
| *B. multivorans* (49) | | | | 0 | 0 | 0 | 2 | 17 | 9 | 13 | 4 | 1 | 1 | 2 | 4 | 4 | 41 | |  |
| *B. pseudomultivorans* (1) | | | | 0 | 0 | 0 | 0 | 0 | 0 | 1 | 0 | 0 | 0 | 0 | 0 | 0 | 1 | |  |
| *B. pyrrocinia* (2) | | | | 0 | 0 | 0 | 0 | 2 | 0 | 0 | 0 | 0 | 0 | 0 | 0 | 0 | 2 | |  |
| *B. seminalis* (1) | | | | 0 | 0 | 0 | 0 | 0 | 1 | 0 | 0 | 0 | 0 | 0 | 0 | 0 | 1 | |  |
| *B. stabilis* (2) | | | | 0 | 0 | 0 | 0 | 0 | 0 | 1 | 1 | 0 | 0 | 0 | 0 | 1 | 1 | |  |
| *B. ubonensis* (1) | | | | 0 | 0 | 0 | 0 | 0 | 0 | 0 | 1 | 0 | 0 | 0 | 0 | 1 | 0 | |  |
| *B. vietnamiensis* (10) | | | | 0 | 0 | 1 | 2 | 2 | 3 | 2 | 0 | 0 | 0 | 0 | 0 | 0 | 10 | |  |
| Bcc Indeterminate (8) | | | | 0 | 0 | 0 | 0 | 3 | 3 | 2 | 0 | 0 | 0 | 0 | 0 | 0 | 8 | |  |
| **Total (150)** | | | | **0** | **0** | **1** | **8** | **57** | **36** | **30** | **13** | **1** | **2** | **2** | **5** | **13** | **132** | |  |
| **Species** | | | **Levofloxacin MIC (µg/mL)** | | | | | | | | | | | | | | | | |
|  | | | | **0.06** | **0.125** | **0.25** | **0.5** | **1** | **2** | **4** | **8** | **16** | **32** | **>32** | **R** | **I** | **S** | |  |
| *B. ambifaria* (4) | | | | 0 | 0 | 0 | 0 | 1 | 0 | 0 | 1 | 0 | 1 | 1 | 3 | 0 | 1 | |  |
| *B. arboris* (2) | | | | 0 | 0 | 0 | 0 | 0 | 0 | 1 | 1 | 0 | 0 | 0 | 1 | 1 | 0 | |  |
| *B. cenocepacia* (33) | | | | 0 | 0 | 0 | 1 | 1 | 5 | 6 | 5 | 3 | 3 | 9 | 20 | 6 | 7 | |  |
| *B. cepacia* (15) | | | | 0 | 0 | 0 | 0 | 0 | 3 | 6 | 2 | 1 | 1 | 2 | 6 | 6 | 3 | |  |
| *B. contaminans* (7) | | | | 0 | 0 | 0 | 0 | 0 | 1 | 3 | 2 | 0 | 1 | 0 | 3 | 3 | 1 | |  |
| *B. diffusa* (1) | | | | 0 | 0 | 0 | 0 | 0 | 1 | 0 | 0 | 0 | 0 | 0 | 0 | 0 | 1 | |  |
| *B. dolosa* (4) | | | | 0 | 0 | 0 | 0 | 0 | 0 | 1 | 0 | 0 | 1 | 2 | 3 | 1 | 0 | |  |
| *B. gladioli* (10) | | | | 0 | 0 | 0 | 3 | 2 | 2 | 1 | 2 | 0 | 0 | 0 | 2 | 1 | 7 | |  |
| *B. multivorans* (48) | | | | 0 | 0 | 0 | 0 | 2 | 14 | 7 | 9 | 7 | 4 | 5 | 25 | 7 | 16 | |  |
| *B. pseudomultivorans* (1) | | | | 0 | 0 | 0 | 0 | 0 | 0 | 0 | 0 | 1 | 0 | 0 | 1 | 0 | 0 | |  |
| *B. pyrrocinia* (2) | | | | 0 | 0 | 0 | 0 | 0 | 0 | 0 | 0 | 0 | 0 | 2 | 2 | 0 | 0 | |  |
| *B. seminalis* (1) | | | | 0 | 0 | 0 | 0 | 0 | 0 | 1 | 0 | 0 | 0 | 0 | 0 | 1 | 0 | |  |
| *B. stabilis* (2) | | | | 0 | 0 | 0 | 0 | 0 | 0 | 0 | 0 | 2 | 0 | 0 | 2 | 0 | 0 | |  |
| *B. ubonensis* (1) | | | | 0 | 0 | 0 | 0 | 0 | 0 | 1 | 0 | 0 | 0 | 0 | 0 | 1 | 0 | |  |
| *B. vietnamiensis* (10) | | | | 0 | 0 | 0 | 0 | 0 | 1 | 1 | 6 | 1 | 0 | 1 | 8 | 1 | 1 | |  |
| Bcc Indeterminate (8) | | | | 0 | 0 | 0 | 0 | 1 | 1 | 1 | 2 | 1 | 1 | 1 | 5 | 1 | 2 | |  |
| **Total (149)** | | | | **0** | **0** | **0** | **4** | **7** | **28** | **29** | **30** | **16** | **12** | **23** | **81** | **29** | **39** | |  |
| Breakpoints for cefepime (susceptible (S) ≤ 8 µg/mL; intermediate = 16 µg/mL; resistant (R) ≥ 32 µg/mL) for *P*. *aeruginosa* were used to assign phenotypes for cefepime and the combination with taniborbactam. Breakpoints for meropenem (susceptible (S) ≤ 4 µg/mL; intermediate (I) = 8 µg/mL; resistant (R) ≥ 16 µg/mL) were used to assign phenotypes to meropenem-vaborbactam. Breakpoints for levofloxacin were as follows (susceptible (S) ≤ 2 µg/mL; intermediate = 4 µg/mL; resistant (R) ≥ 8 µg/mL). *taniborbactam was tested at a fixed concentration of 4 µg/mL, while vaborbactam was tested at a fixed concentration of 8 µg/mL. | | | | | | | | | | | | | | | | | |  |  |

Table S3 Agar dilution susceptibility testing results for quality control strains.

| Strains | *bla* genes | cefepime | cefepime-  taniborbactam* | meropenem | meropenem-vaborbactam* | levofloxacin |
| --- | --- | --- | --- | --- | --- | --- |
|  | MIC Ranges (µg/mL) | | | | | |
| *K. pneumoniae* ATCC 700603 | *bla*_SHV-18_  *bla*_OXA-2_  Mutations in  *ompk35* and *ompk37* | 0.5-1 | 0.25-0.5 | 0.06 | 0.06 | 0.5-1 |
| *K. pneumoniae* ATCC BAA-1705 | *bla*_KPC-2_  *bla*_TEM_  *bla*_SHV_ | 8-32 | 0.125-0.5 | 4-16^#^ | 0.06 | >32 |

*Taniborbactam was tested at a fixed concentration of 4 µg/mL, while vaborbactam was tested at a fixed concentration of 8 µg/mL.

^#^Values represent the range for 16 independent experiments; in two experiments the value dropped to 4 µg/mL but went back to 8 µg/mL and was 8 µg/mL for 10/16 experiments.
